# Mitochondrial DNA Hypomethylation Is a Biomarker Associated with Induced Senescence in Human Fetal Heart Mesenchymal Stem Cells

**DOI:** 10.1155/2017/1764549

**Published:** 2017-04-06

**Authors:** Dehai Yu, Zhonghua Du, Lingling Pian, Tao Li, Xue Wen, Wei Li, Su-Jeong Kim, Jialin Xiao, Pinchas Cohen, Jiuwei Cui, Andrew R. Hoffman, Ji-Fan Hu

**Affiliations:** ^1^Stem Cell and Cancer Center, The First Bethune Hospital, Jilin University, Changchun, Jilin 130061, China; ^2^Stanford University Medical School, Palo Alto Veterans Institute for Research, Palo Alto, CA 94304, USA; ^3^Leonard Davis School of Gerontology, University of Southern California, Los Angeles, CA 90089, USA

## Abstract

*Background*. Fetal heart can regenerate to restore its normal anatomy and function in response to injury, but this regenerative capacity is lost within the first week of postnatal life. Although the specific molecular mechanisms remain to be defined, it is presumed that aging of cardiac stem or progenitor cells may contribute to the loss of regenerative potential.* Methods*. To study this aging-related dysfunction, we cultured mesenchymal stem cells (MSCs) from human fetal heart tissues. Senescence was induced by exposing cells to chronic oxidative stress/low serum. Mitochondrial DNA methylation was examined during the period of senescence.* Results*. Senescent MSCs exhibited flattened and enlarged morphology and were positive for the senescence-associated beta-galactosidase (SA-*β*-Gal). By scanning the entire mitochondrial genome, we found that four CpG islands were hypomethylated in close association with senescence in MSCs. The mitochondrial COX1 gene, which encodes the main subunit of the cytochrome c oxidase complex and contains the differentially methylated CpG island 4, was upregulated in MSCs in parallel with the onset of senescence. Knockdown of DNA methyltransferases (DNMT1, DNMT3a, and DNMT3B) also upregulated COX1 expression and induced cellular senescence in MSCs.* Conclusions*. This study demonstrates that mitochondrial CpG hypomethylation may serve as a critical biomarker associated with cellular senescence induced by chronic oxidative stress.

## 1. Introduction

The adult mammalian heart has traditionally been viewed as a nonregenerative organ as it retains very minimal regenerative potential. Following cardiac injury, as in the case of myocardial infarction, the heart fails to replace the vast majority of lost or damaged cardiomyocytes. Instead, the heart heals with scar tissue, leading to a contractile defect of the organ and ultimately heart failure [1]. However, the hearts of one-day-old mouse retain full regenerative potential and are able to restore normal anatomy and function after cardiac injury [2]. This regenerative capacity of the neonatal mouse heart, however, becomes lost within the first week of postnatal life. Decreases in cardiac stem or progenitor cells could be one of the determining factors for the loss of regenerative capacity in this aging process [3].

Mesenchymal stem cells (MSCs) have attracted great interest as a promising regenerative therapeutic for many human diseases, primarily because of their capacity for self-renewal, multilineage differentiation, and immune modulation [9–13]. MSCs derived from fetal heart (c-MSCs, HMSCs) have the potential to differentiate into cardiomyocytes, endothelial cells, and smooth muscle cells [3, 14–16]. Engraftment of fetal cardiac MSCs improves cardiac function and can repair myocardium in a rat model of myocardial infarction [15]. However, to serve as a useful regenerative therapy, MSCs isolated from patients need to be expanded ex vivo [17]. MSCs have a limited lifespan in cell culture, and after a few passages of expansion, the cells enter the senescent state, leading to the reduction in self-renewal ability and differentiation potential [18, 19]. Moreover, the self-renewal potential of MSCs is significantly reduced with aging. Like many primary cells in culture, MSCs show decreased proliferation and increased apoptosis as the age of the donor animal or human [20–24]. As MSCs approach senescence, their proliferation slows significantly, leading to a decrease in differentiation potential [25]. Therefore, it is critical to understand the mechanisms that control replicative senescence in MSCs.

Replicative senescence, a process that places cells in permanent proliferative arrest in response to various stressors, is a potentially important contributor to aging and age-related disease. It is clear that cellular senescence is associated with an array of epigenetic modifications that may be responsible for changes in gene expression that ultimately lead to catabolic and degenerative processes. Mitochondrial DNA (mtDNA) damage has long been implicated in the aging process [26]. A number of mitochondrial signaling pathways can induce cellular senescence [27]. However, the role of mitochondrial epigenetics has largely been unexplored. In fact, the importance and relevance of mitochondrial epigenetics in aging has been controversial [28], primarily because of the difficulty of studying the relatively small mitochondrial genome in the context of the far larger nuclear genome.

Methylation of mtDNA in a variety of tissues varies with aging, disease states, environmental exposure, and certain drugs. In addition to 5-methylcytosine, a so-called “sixth base,” 5-hydroxymethylcytosine has also been identified in mtDNA, where its abundance changes during aging independently of 5-methylcytosine levels [29]. It has been suggested that mtDNA methylation might become a next-generation biomarker for aging [30]. Currently, we know very little about the role of mtDNA methylation in the aging-related dysfunction of cardiac stem or progenitor cells. To gain insight regarding the role of mitochondrial epigenetics in senescence, we induced senescence in MSCs cultured from human fetal heart tissues and examined epigenetic mechanisms that may be associated with cellular aging in MSCs.

## 2. Materials and Methods

### 2.1. Isolation of MSCs

Isolation of MSCs from fetal heart tissues was performed following the method as described previously [3, 16] with some modifications. Briefly, fetal heart tissues from days 45–67 of embryos were obtained from Human Tissue Network (Central Laboratory for Human Embryology Tissue, University of Washington, WA) in phosphate-buffered saline (PBS). Upon arrival, the tissues were minced with a razor into small pieces. To avoid destroying the cardiac stem cell niche, we did not digest the tissue with collagenase. Instead, the minced heart tissues were directly seeded on a 10 cm plate with a small amount of DMEM (2-3 ml) containing 10% fetal calf serum (FCS) and antibiotics and were cultured at 37°C in a 5% CO_2_ humidified atmosphere. After the heart tissue became attached to the plate, a small amount of fresh medium was added to the plate without disturbing the tissues on a daily basis. After 5–7 days, MSCs began to migrate out from the heart explants and the MSCs were collected for further expansion. MSCs at passages 3–5 were used for analysis. MSCs from fetal skin tissues were isolated using the same approach and were used in parallel with HMSCs in the study. The experimental protocol was approved by the Institutional Review Board/Stem Cell Research Oversight Panel at Stanford University.

### 2.2. Characterization of MSCs

The MSCs cultured from fetal heart (HMSCs) were characterized by flow cytometry using stem cell markers as previously described [31]. Cells were collected at a concentration of 1 × 10^6^ cells/ml in phosphate-buffered saline (PBS) containing 0.1% BSA. Cells were incubated at 4°C with antibodies against MSC-markers (CD90, CD73, and CD105), hematopoietic cell markers (CD45, CD34, CD14, and CD19), and receptors for extracellular matrix (CD29, CD44) and major histocompatibility (HLA-DR) (all from BD Biosciences, CA). Thirty minutes after antibody incubation, cells were washed and suspended in 300 *μ*L PBS. Flow cytometry was performed using FACSCAria III Cell Sorter (BD Biosciences, CA) [32–35].

### 2.3. Differentiation of MSCs

The differential potential of the isolated MSCs into adipogenic and osteogenic lineages was performed as described previously [32–35]. After induction, cells were stained with the Oil Red O and Alizarin Red (Sigma, USA) to detect the presence of neutral lipid vacuoles in differentiated adipocytes and calcium deposition in osteocytes, respectively.

### 2.4. Induced Senescence in MSCs

To mimic the replicative senescence seen in MSCs [36, 37], we adopted a new approach by chronically exposing cells to low oxidative stress and a low serum environment. Specifically, HMSCs at passages 3–5 at approximately 50% confluence were continuously exposed to low concentration of hydrogen peroxide (50 *µ*M H_2_O_2_) in DMEM supplemented with 5% FBS. Using this approach, MSCs exhibited a typical senescence-like morphology after 2-3 passages. Cells were collected for RNA-Seq analysis (Beijing Honor Tech Co., Ltd., Beijing, China).

### 2.5. Staining of Senescence-Associated *β*-Galactosidase

Cellular senescence was quantitated by measuring the activity of SA-*β*-Gal as previously described [38, 39]. MSCs were collected and fixed using 3% formaldehyde for 3–5 min at room temperature. After washing with PBS, cells were incubated at 37°C with freshly prepared senescence-associated SA-*β*-Gal staining solution: 1 mg/ml 5-bromo-4-chloro-3-indolyl P3-D-galactoside (X-Gal), 40 mM citric acid/sodium phosphate, pH 6.0, 5 mM potassium ferrocyanide, 5 mM potassium ferricyanide, 150 mM NaCl, and 2 mM MgCl2. After overnight incubation, cells with the SA-*β*-Gal staining were assessed using a microscope-mounted camera.

### 2.6. Detection of Telomere Length in Aging Cells

The relative length of telomeres was estimated by quantitative PCR as previously reported [40, 41]. Briefly, genomic DNA was extracted with Qiagen DNeasy Blood & Tissue Kit (Qiagen, CA, USA). After dilution, 35 ng/*μ*l DNA was heated at 95°C for 5 min and chilled on ice for 5 min. The DNA samples were placed in a 20 *μ*l real-time qPCR reaction system containing 10 *μ*l 2 × SYBR premixed buffer (Roche, Shanghai, China), 2 *μ*l forward and reverse primers. The sequence of primers includes (1) telomere forward: 5′-GGTTTTTGAGGGTGAGGGTGAGGGTGAGGGTGAGGGT-3′ (100 nM) and reverse: 5′-TCCCGACTATCCCTATCCCTTCCCTATCCCTATCCCTA-3′ (300 nM); and (2) *β*-globin forward: 5′- GCTTCTGACACAACTGTGTTCACTAGC-3′ (150 nM) and reverse: 5′-CACCAACTTCATCCACGTTCACC-3′ (150 nM). The PCR amplification process was one cycle at 95°C 10 min and 40 cycles at 95°C for 15 s, 56°C for 30 s, and 72°C 30 s (ABI SepOnePlus, Beijing, China). The telomere length was estimated by the relative ratio between the copies of telomere and the copies of *β*-globin (T/S). T/S value is calculated by 2^−ΔΔCt^ [40, 41].

### 2.7. Gene Expression of the Senescence and the Cardiac Development Pathways

Comparison of the senescence and cardiac development pathway genes was performed by RNA-Seq [42]. Total RNA was isolated using Qiazol (Qiagen, CA) and used for indexed library preparation using Illumina's TruSeq RNA Sample Prep Kit v2. Libraries were sequenced using a HiSeq4000 (Illumina) and yielded approximately 34 million reads with a length of 150 bp per sample. Gene counts were normalized to the values of Reads Per Kilobase of transcript per Million mapped reads (RPKM). KEGG pathways were selected as significantly regulated if the corrected *P* values were smaller than 0.05 (Beijing Honor Tech Co.,Ltd., Beijing, China).

### 2.8. Measurement of mtDNA Methylation by COBRA

Mitochondrial and total cellular DNAs were extracted by DNeasy Blood & Tissue Kit (Qiagen, CA) and treated by sodium bisulfite using EZ DNA Methylation™ Kit (Zymo, CA), following the protocol provided by the manufacturer. Initially, we obtained mtDNA from isolated mitochondria and treated the DNA with sodium bisulfite for cloning sequencing. Later, we found that the protocol could be greatly simplified by simply using total genomic DNA with PCR primers that are specific for mitochondrial DNA.

Bisulfite-treated DNA was amplified by polymerase chain reaction (PCR) under liquid wax in a 6 *µ*l reaction containing 2 *µ*l of 3 × Klen-Taq I Mix, 2 *µ*l template DNA, and 1 *µ*l of each 2.5 *µ*M primer. After incubation at 95°C for 5 min, DNA was amplified by 38 cycles of 95°C for 20 s, 62°C for 20 s of annealing, and 72°C for 20 s of extension and finally with extension at 72°C for 2 min. Methylation PCR primers sequences are listed in Table S1 (see Table S1 in the Supplementary Material available online at https://doi.org/10.1155/2017/1764549).

The status of mtDNA methylation was determined by restriction enzyme digestion. PCR DNAs were digested by Taq I at 65°C for 2 h or HpyCH4IV at 37°C for 2 h and separated on 3% agarose gel. Taq I recognizes the TCGA site and HpyCH4IV digests the ACGT site. After treatment with sodium bisulfate, unmethylated cytosines were converted to uracils [43, 44]. As a result, methylated mtDNA will be digested by the restriction enzymes. In contrast, unmethylated mtDNAs will be converted to TTGA and ATGT, which are not digested by Taq I and HpyCH4IV, respectively. The methylated and unmethylated bands were scanned for quantitation.

### 2.9. Knockdown of DNMTs by RNA Interference Lentiviruses

Two different RNA interference oligonucleotides targeting each DNMT gene were driven by H1 and U6 promoters, respectively, and were jointly subcloned into pGreen-puro lentiviral vector by PCR. The oligonucleotide sequences targeting DNMT1, DNMT3a, and DNMT3b are as follows: DNMT1 (A): 5′-GCCCAATGAGACTGACATCAA-3′; DNMT1 (B): 5′-GGAACCAAGCAAGAAGTGA-3′; DNMT3a (A): 5′-AGCGGGCAAAGAACAGAAG-3′; DNMT3a (B): 5′-CCAGATGTTCTTCGCTAATAA-3′; DNMT3b (A): 5′-CCTGTCATTGTTTGATGGCAT-3′; DNMT3b (B): 5′-CCATGCAACGATCTCTCAAAT-3′. The scramble control sequence is 5′-GCTTCAATTCGCGCACCTA-3′.

For viral packaging, 293 T cells were transfected with 2 *µ*g of each lentiviral expression construct. Transfections were done in six-well plates using Lipofectamine 2000 (Invitrogen, USA). Viral supernatants were collected at 24 and 48 h after transfection. After addition of polybrene (8 *µ*g ml^−1^), the supernatants were placed on the cultured HMSCs cells. Cells were transfected twice to increase transfection efficiency.

### 2.10. RT-PCR Analysis

RT-PCR was used to quantitate the expression of genes related to senescence. Total RNA was extracted by TRIzol reagent (Sigma, MO) and was converted to cDNA by reverse transcription reaction as previously described [45, 46]. We designed PCR primers as follows: *β*-*actin*: 5′-CAGGTCATCACCATTGGCAATGAGC-3′ (forward) and 5′-CGGATGTCCACGTCACACTTCATGA-3′ (reverse);* caveolin-1*: 5′-TCCCATCCGGGAACAGGGCAACAT-3′ (forward) and 5′-GTCCCTTCTGGTTCTGCAATC-3′ (reverse);* P16*: 5′-CGGATAATTCAAGAGCTAACAGGT-3' (forward) and 5′-GGCCTCCGACCGTAACTATTCGGT-3′ (reverse);* P21*: 5′-GTGGACCTGTCACTGTCTTGTAC-3′ (forward) and 5′-GCTTCCTCTTGGAGAAGATCAGC-3′ (reverse); apolipoprotein: 5′-GGTCTCWGACAATGAGCTCCA-3 (forward) and 5′-TCCCAGAGGGCCATCATGGTC-3′ (reverse);* COX1*: 5′-CAGCATGCCCCAGGATTTGTC-3′ (forward) and 5′-CAKGTCCTGCTCCAGGGCAGC-3′ (reverse, K = G/T). The PCR amplification was composed of 1 cycle at 95°C for 5 min and 33 cycles at 95°C 20 s, 62°C 15 s, and 72°C 15 s and ending with an extension cycle at 72°C 5 min.

### 2.11. Measurement of COX1 Enzyme Activity

COX1 activity was determined by cellular staining for cytochrome C oxidase. MSCs (1 × 10^6^) were plated in 6-well plate and were cultured for 24 hrs. Cells were rinsed 3 times with PBS and were dried in air. Cells were incubated for 15 min at RT in the preincubation medium (50 mM Tris-HCl, pH 7.6; 0.29 M sucrose; 2.2 mM cobalt chloride) and were rinsed with buffer I (0.1 M sodium phosphate pH 7.6; 10% sucrose). Cells were incubated for 4 hrs at 37°C in 10 ml incubation medium (pH 7.4, 0.1 M sodium phosphate pH 7.6, 10% sucrose, 10 mg cytochrome C (Sigma, MO), 10 mg DAB (3,3′-diaminobenzidine) hydrochloride (Sigma, MO), and 2.0 mg catalase (Sigma, MO)). Cells were rinsed once with buffer I and 5 min with PBS, washed with H_2_O for 5 min, and observed under microscope.

### 2.12. Statistical Analysis

Data were analyzed using SPSS software (version 16.0; SPSS, Inc., IL). Student's *t*-test or one-way ANOVA (Bonferroni test) was used to compare statistical differences for variables among treatment groups. The data were expressed as mean ± SD. All experiments were performed in triplicate, and results were considered statistically significant at *P* < 0.05.

## 3. Results

### 3.1. Multilineage Differentiation of Fetal Heart Mesenchymal Stem Cells

MSCs isolated from different tissues, despite their molecular congruence, exhibit strong biases in gene and protein expression, pathway activity, and lineage differentiation, suggesting the presence of “molecular memory of tissue origin” [14]. These conserved organ-specific functions may potentially render them more appropriate as cellular therapeutic agents for their organ of origin, particularly in “*in situ* reprogramming” or “*in situ* differentiation” models. Of note, the murine neonatal heart can regenerate and restore damaged sections resulting in the restoration of normal anatomy and function without scar formation, but this capacity is lost after one week of age [2]. We were interested to learn if epigenetic alterations in mitochondria DNA were involved in this aging process.

**Figure 1:**
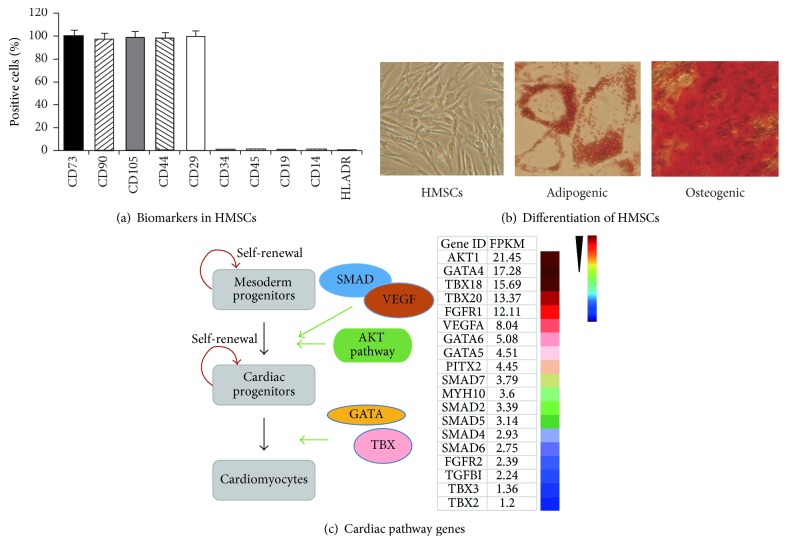
Characterization of human fetal heart-derived mesenchymal stem cells (HMSCs). (a) The profile of stem cell markers in cultured HMSCs. Immunophenotypes of MSCs were determined by flow cytometry using labeled antibodies specific for the indicated human surface antigens. (b) Differentiation potential of HMSCs. Cells were stained by Alizarin Red for calcium deposits during osteogenic differentiation. Adipogenic differentiation was detected by Oil Red O staining (200x). (c) The “molecular memory of cardiac origin” of HMSCs. Left panel: schematic diagram of the published cardiac stem cell pathways [4–8]. Right panel: expression of pathway genes in HMSCs. Total RNAs were isolated from HMSCs for RNA-Seq using a HiSeq4000 (Illumina). Colors represent from high (red) to low (blue) expression based on normalized FPKM values for each gene.

To define the potential epigenetic mechanisms underlying this cardiac aging, we cultured MSCs from human fetal heart tissues, which are presumed to maintain full regenerative potential. Adherent MSCs grew ~8–12 days after the initial tissue seeding and were collected for phenotypic analysis using flow cytometry. We found that stem cell markers were universally expressed in isolated MSCs, including CD105, CD73, CD90, CD44, and CD29 (Figure 1(a)). Negative stem cell markers, including CD45, CD34, CD14, CD19, and HLA-DR, were expressed at very low levels. The isolated HMSCs could be differentiated into adipogenic and osteogenic lineages (Figure 1(b)). These data suggest that the MSCs cultured from fetal heart exhibited the potential of multilineage differentiation as previously reported [3, 14, 16].

As tissue MSCs exhibit strong “molecular memory of tissue origin” [14], we used RNA-Seq to examine the pathway genes that are associated with cardiac development [4–8]. We found that genes involved in cardiac programing were also expressed in HMSCs, including AKT pathway, GATA family, and TBX family genes (Figure 1(c)). These data suggest that these HMSCs may serve as an appropriate model to study cardiac aging.

### 3.2. Induction of Chronic Senescence in Mesenchymal Stem Cells

Two types of induced senescence have commonly been used to study aging* in vitro*, including replicative senescence (RS) and stress-induced premature senescence (SIPS) [47–49]. RS is characterized by progressive telomere shortening, which occurs at every cell division. Although it is an excellent mimic of the natural aging process, induction of senescence is very time-consuming. On the other hand, SIPS is induced by exposure to subcytotoxic stress, and cells undergo premature senescence without telomere shortening. SIPS is the most frequently used model in studying aging. However, cellular toxicity is encountered in this model, particularly by high concentrations of hydrogen peroxide. Therefore, SIPS may not be an appropriate model of* in vivo* pathophysiological aging in cardiac stem cells.

**Figure 2:**
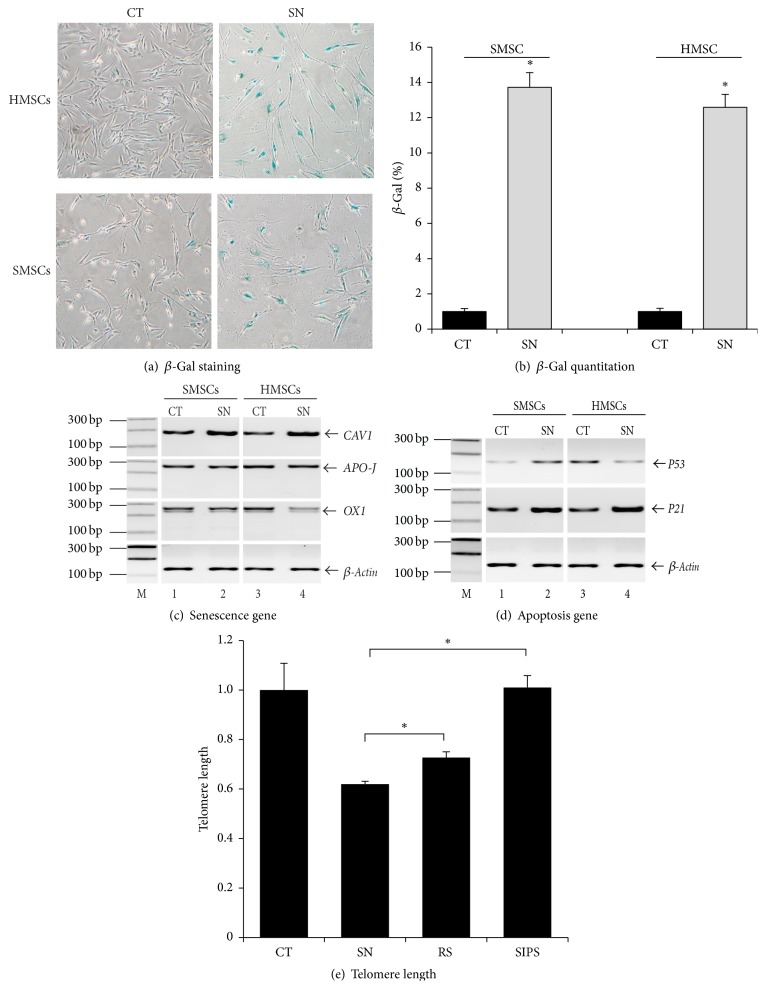
Induction of premature senescence in MSCs. (a) Induced senescence in MSCs. Senescence was induced by continuous exposure of HMSCs (heart-derived MSCs) and SMSCs (skin-derived MSCs) to a low dose of H_2_O_2_ (50 *µ*M) and low serum (5% FBS) in the culturing medium. CT: control HMSCs treated with PBS; SN: senescent HMSCs treated with 50 *µ*M H_2_O_2_ for 14 d (×10). (b) Quantitation of *β*-Gal cells. Cells were counted under microscopy. The results are expressed as the mean ± standard deviation of *β*-Gal positive cells per field. ^*∗*^*P* < 0.05 as compared with the PBS control. (c) Senescent-related genes. Senescent HMSCs were harvested and RNA were extracted. RT-PCR was carried out to amplify senescence-related genes, including caveolin-1, apolipoprotein J, and OX 1. (d) Apoptosis-related genes. Expression of p53 and p21 genes was measured by RT-PCR. (e) Telomere length in senescent HMSCs. The relative length of telomere was estimated by qPCR as the ratio between the copies of telomere and the copies of *β*-globin (T/S). ^*∗*^*P* < 0.05 as compared with the replicative senescence and high H_2_O_2_ groups.

In this study, we established a novel model by combining the advantages of both RS and SIPS. In this model, we facilitated the occurrence of replicative senescence by a new “two-hit” approach. Fetal heart-derived MSCs (HMSCs) were exposed simultaneously to low doses of hydrogen peroxide (50 *µ*M) and to low serum (5%) in cell culture. It was presumed that exposure to low dose of hydrogen peroxide would generate much less toxicity than that usually encountered in SIPS. Meanwhile, low serum exposure would accelerate the development of senescence. After combined exposure for 2-3 passages, we found that HMSCs exhibited flattened morphology and decreased cell proliferation. The treated MSCs stained positive for senescence-associated beta-galactosidase (SA-*β*-Gal) activity (Figures 2(a) and 2(b)).

We then used PCR to quantitate senescence-related genes that have been previously reported [38, 39, 50]. We found that the caveolin-1 gene (CAV1) was upregulated in senescent MSCs (Figure 2(c)), although we did not detect a significant change in APO-J and OX1. P21 was also upregulated in parallel with cell senescence (Figure 2(d)). Future studies are needed to clarify the time point from the senescence-inducing stimulus when p53 and p21 start to be upregulated.

To further characterize the senescent cells, we measured telomere length using quantitative PCR. By comparing telomere length among the three models, it was clear that the senescent cells in our model had the telomere shortening similar to that seen in replicative senescent cells (Figure 2(e)). As expected, there was no significant change in telomere length in cells that were treated with high dose of hydrogen peroxide. In addition, using RNA-Seq we found that the pathways previously identified in replicative senescent cells [42, 51] were also activated in our senescent cells (Figures S1-S2). Together, these data suggest that our model carries a phenotype that is prone to replicative senescence.

### 3.3. Cellular Senescence Is Associated with Differential Methylation of mtDNA CpG Islands

**Figure 3:**
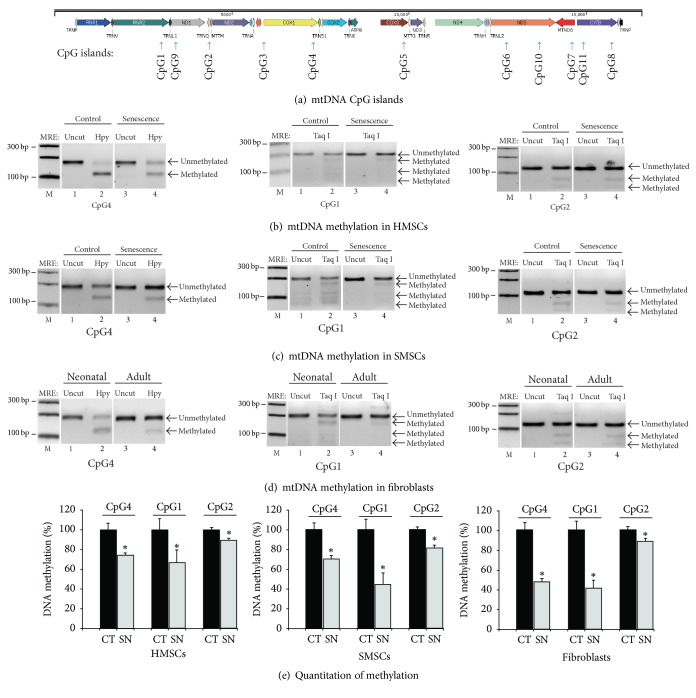
Altered mtDNA methylation in senescent MSCs. (a) Schematic diagram of the mitochondrial genes and the location of CpG islands. In order to detect mtDNA methylation in senescent cells, we designed 11 pairs of methylation-specific primers located on different genes on mitochondrion. (b) Comparison of mtDNA methylation between the control and senescent HMSCs. mtDNA methylation was measured by combined bisulfite restriction analysis (COBRA). PCR products from mtDNA of control and senescent HMSCs were digested by TaqI or HpyCH4IV (HPY) to separate the unmethylated and methylated DNAs. Taq I and HpyCH4IV recognize and digest the methylated ACGT and TCGA sites, respectively. After treatment with sodium bisulfate, unmethylated cytosines were converted to uracils, and the TTGA and ATGT sites are not digested by these two enzymes. After digestion, unmethylated and methylated DNA were separated on 3% agarose gels. Only the data for CpG islands 4, 2, and 1 are presented here. (c) Differential mtDNA methylation between the control and senescent SMSCs. (d) Altered mtDNA methylation in human neonatal and adult fibroblasts. (e) Quantitation of mtDNA CpG methylation. The methylated and unmethylated bands were scanned. The status of CpG methylation was calculated as the relative percentage of DNA methylation using the untreated MSCs as 100. ^*∗*^*P* < 0.05 as compared with that in untreated MSC control cells. Note the decrease in mtDNA methylation in senescent MSCs.

To depict the epigenetic mechanism underlying cellular senescence, we used sodium bisulfite sequencing to assess DNA methylation in a total of 11 CpG islands throughout the mitochondrial genome (Figure 3(a)). Using restriction enzymes to distinguish the methylated and unmethylated CpGs, we found that the mitochondrial genome was primarily unmethylated. Three CpG islands exhibited considerable mtDNA hypomethylation in senescent HMSCs, including CpG islands 4, 1, and 2 (Figure 3(b)). Similar mtDNA methylation pattern was also observed in SMSCs (Figure 3(c)). The remaining CpG islands, however, showed fewer differences between the control and senescent MSCs (Figures S3–S5). We also noticed the difference in mtDNA methylation in three CpG islands (4, 2, and 1) between neonatal and adult skin fibroblasts (Figure 3(d)).

The degree of mitochondrial DNA methylation was quantitated by scanning the PCR band density. Clearly, significant mtDNA hypomethylation was observed in cells following senescence (Figure 3(e)).

### 3.4. Differential mtDNA Methylation Affects COX1 Expression

**Figure 4:**
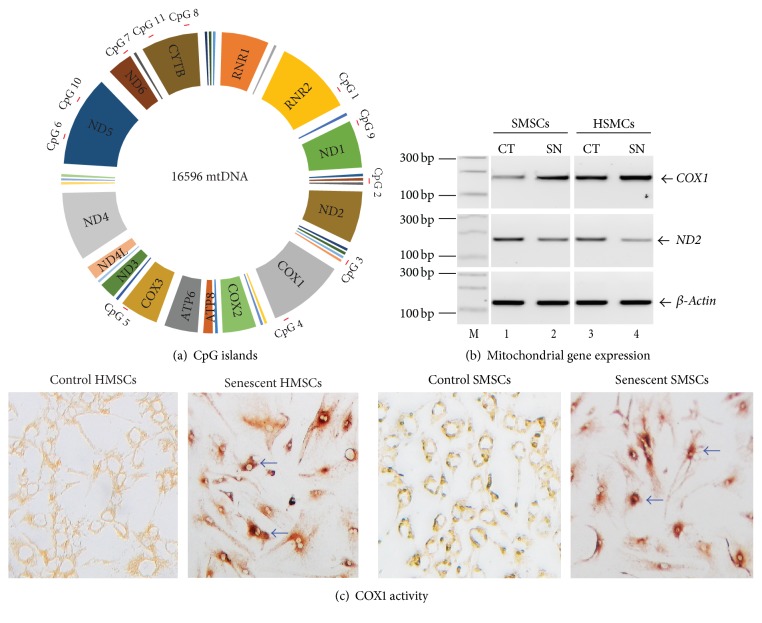
Alteration of mitochondrial genes during cellular senescence. (a) Location of CpG islands in the mitochondrial genome. (b) Altered gene expression of mitochondrial COX1 and ND2 genes in senescent MSCs. (c) The enzyme activity of COX1 in control and senescent MSCs.

CpG island 4 is located within the 3′-region of the mitochondrial COX1 gene (Figure 4(a)), which encodes cytochrome c oxidase I, a key enzyme in aerobic metabolism. As the degree of mtDNA methylation at CpG 4 declines following senescence, we suspected that COX1 expression would be affected by this epigenetic regulation. We used RT-PCR to semiquantitate COX1 and found that a significant upregulation of COX1 was associated with senescence (Figure 4(b)). ND2, a second mitochondrial gene located downstream of CpG island 2, was slightly decreased in senescent MSCs. Similarly, the activity of the COX1 enzyme was also increased in senescent MSCs cultured from fetal heart and skin tissues (Figure 4(c)). Thus, altered mtDNA methylation may be accompanied by subtle changes in the activity of mitochondrial enzymes.

### 3.5. Knockdown of DNA Methyltransferases Upregulates Mitochondrial COX1

**Figure 5:**
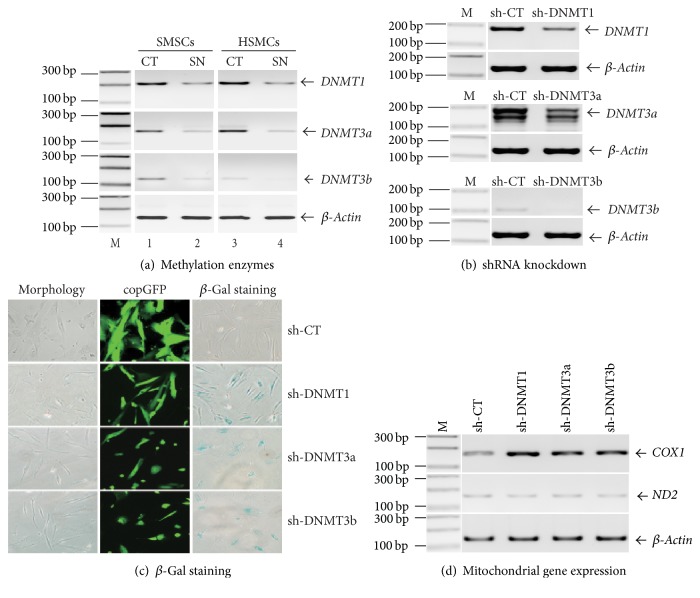
Knockdown of DNMTs induces senescence in MSCs. (a) Downregulation of DNMTs in senescent MSCs. After induction of cellular senescence, cells were harvested and the expression of DNMTs was determined by semiquantitative PCR. (b) Knockdown of DNMTs by shRNAs. Lentiviruses containing DNMT shRNAs (sh-DNMT1, sh-SNMT3a, and sh-DNMT3b) or scramble control (sh-CT) were transduced into HMSCs. After 72 h, transduction efficiency was assessed by the observation of GFP positive cells. Cells were harvested and RNA were extracted, and the expression of DNMTs was determined by RT-PCR. (c) Induction of cellular senescence by DNMT-shRNA knockdown. Left panel: HMSCs morphology taken 7 days after DNMT-shRNA transduction. Sh-CT: shRNA scramble control; sh-DNMTs: HMSCs were transducted by lentiviruses containing DNMT1, DNMT3a, and DNMT3b shRNAs. Middle panel: lentiviral transduction efficiency as shown by copGFP fluorescence of the shRNA vector. Right panel: senescent-associated *β*-Gal staining. After DNMT-shRNA knockdown, MSCs were stained for *β*-Gal activity. Note the occurrence of senescence in DNMT-knockdown MSCs in the absence of peroxide treatment. (d) Expression of COX and ND2 in DNMT-shRNA-treated MSCs. *β*-Actin was used as the internal control for PCR reaction. Cox1 was upregulated in DNMT-knockdown MSCs in parallel with cellular senescence.

Since mtDNA becomes hypomethylated during senescence, we examined the role of three DNA methyltransferases (*DNMT1, DNMT3A,* and* DNMT3B*) that control DNA methylation. We found that all three enzymes were downregulated in senescent MSCs (Figure 5(a)).

To confirm the role of DNA methyltransferases in senescence, we knocked down the enzymes with shRNAs (Figure 5(b)). Interestingly, knockdown of these three enzymes induced senescence and inhibited cell proliferation in MSCs (Figure 5(c)), in parallel with the upregulation of COX1 (Figure 5(d)).

## 4. Discussion

The mechanisms underlying the loss of regenerative potential of the fetal heart during postnatal life remain to be illustrated. In this study, we cultured multipotent MSCs from human fetal heart tissues and used a chronic oxidative stress/low serum approach to induce typical senescence. By scanning the status of CpG methylation in the whole mitochondrial genome, we demonstrated considerable mtDNA hypomethylation following senescence in MSCs. COX1, encoding the main subunit of the cytochrome c oxidase complex, was significantly upregulated in senescent MSCs. Knockdown of three DNA methyltransferases (DNMT1, DNMT3a, and DNMT3B) also upregulated COX1 and induced senescence in MSCs. Together, our data suggest that mitochondrial CpG hypomethylation may be a useful biomarker in association with cellular senescence in MSCs.

Significant progress has been made in deciphering the regulatory pathways that control cellular senescence. There are two distinct pathways that lead to the development of cellular senescence [47, 52, 53]. Replicative senescence (RS) depends on the dysfunction in biological clock that is caused by progressive shortening of repetitive DNA sequences (TTAGGG) in telomeres that cap the ends of each chromosome. This shortening eventually triggers DNA damage and initiates a program of cell cycle arrest. In contrast, stress-induced premature senescence (SIPS), although sharing many cellular and molecular features as those undergoing replicative senescence, is telomere-independent. In SIPS, ROS induces cellular dysfunctions, playing a critical role in age-related diseases [54]. In the SIPS senescence model, oxidative stress is often used as the inducer of SIPS [55]. The cells at early passages were exposed once or several times to acute, sublethal oxidative stress, such as H_2_O_2_ [56, 57]. In order to capture the characteristics of both replicative senescence and SIPS, we adopted a modified approach by exposing MSCs to a low concentration of H_2_O_2_ and a low serum supply. Using this strategy, we induced typical cellular senescence in MSCs within a short period of time, usually ~2-3 passages. Significantly, these senescent cells exhibit shortened telomeres. RNA-Seq confirms that the senescence is accompanied by the activation of the same pathways as seen in replicative senescence. Thus, this approach may provide an ideal model to study cellular senescence in MSCs. Future studies will be needed to comprehensively compare the gene- and protein-expression profiles of this model with two well-established SIPS and RS models.

Replicative senescence in culture expansion of MSC appears to be epigenetically controlled by DNA methylation and repressive histone marks at the genome DNA level [58]. However, epigenetic regulation of the mitochondrial genome in the aging of MSCs is poorly defined [30, 59]. Age-associated accumulation of mtDNA mutations has been proposed to be responsible for the age-associated mitochondrial respiration defects found in elderly human subjects. Aging phenotypes are reversible and controlled by epigenetic regulation. Reprogramming of elderly fibroblasts restores age-associated mitochondrial respiration defects [60]. We measured the status of mtDNA methylation in senescent MSCs cultured from human fetal heart tissues. Overall, the mtDNA is generally hypomethylated in senescent MSCs. This is in agreement with the extent of mtDNA methylation reported in human blood samples and lung tissues [61]. However, we noticed considerable changes in mtDNA methylation during cellular senescence. Particularly, mtDNA at CpG island 4 became more hypomethylated. This senescence-related mtDNA demethylation was also observed in fibroblasts cultured from human adult skin as compared with those from neonatal skin tissues. Clearly, the mitochondrial genome undergoes epigenetic alterations in cellular senescence.

Accompanying the alteration of mtDNA methylation, the mitochondrial COX1 gene, which includes CpG island 4, was upregulated in all aged MSCs, as shown at both mRNA and protein enzyme levels. COX1 encodes cytochrome c oxidase I, the main subunit of the cytochrome c oxidase complex, which is a key enzyme in aerobic metabolism [62]. The proton pumping heme-copper oxidase represents the terminal, energy-transfer enzyme of respiratory chains in both prokaryotes and eukaryotes. Mitochondria generate adenosine 5′-triphosphate (ATP) but also produce potentially toxic reactive oxygen species (ROS) [59]. Gradual mitochondrial dysfunction is observed to accompany aging. Continuous accumulation of mtDNA damage may play a causal role in the aging process. However, it is unclear if the upregulated COX1 in our model is functionally involved in the initiation of senescence or it is just a sequela of the aging process.

Currently, we know little about the role that mtDNA methylation plays in the regulation of gene activity in our senescence model. For example, CpG island 4 is located in the 3′-region of the mitochondrial COX1 gene, rather than in the D-loop promoter region, where we might expect a CpG island to exert epigenetic control. However, several studies have suggested a role for gene body DNA methylation in gene regulation [63–65]. In addition, we also found that knockdown of DNA methyltransferases using shRNA induced the upregulation of COX1 in MSCs (Figure 5(d)), in parallel with cellular senescence (Figure 5(c)), in support of a role of mtDNA hypomethylation in the regulation of COX1. DNMT1, the most abundant DNA methyltransferase in mammalian cells, is the key methyltransferase required for the maintenance of DNA methylation in mammals. It predominantly catalyzes methylation at hemimethylated CpG di-nucleotides [66]. Homozygous deletion of DNMT1 is lethal for mouse embryos at 10-11 days of gestation [67]. Both DNMT3a and DNMT3b, on the other hand, are* de novo* methyltransferases [66]. All three enzymes are required for the establishment, maintenance, and erasure of epigenotypes, including genomic imprints, in mammalian development [68, 69]. In our induced senescence model, these three DNMTs were downregulated in senescent MSCs (Figure 5(a)). Importantly, knockdown of DNMTs by shRNA in the absence of the oxidative stress also induced cellular senescence in MSCs (Figure 5(c)). Our data thus suggest that all three DNMTs are involved in senescence of MSCs. It should be noted that knockdown of DNMTs by shRNA also induces global DNA demethylation, including genomic DNAs. In addition, it is not clear whether mtDNA hypomethylation is a specific biomarker for our model. Particularly, we do not know if it also might occur in the replicative senescence and SIPS models. Future studies need to be performed by comparing the epigenetics of mitochondrial DNA in these models. We can learn more about epigenetic control using site-specific de novo DNA methylation or demethylation that can be induced by the CRISPR Cas9-Sss1 epigenetic regulators [43].

## 5. Conclusions

In summary, by scanning DNA methylation in mitochondrial genome, we demonstrate the association of aberrant epigenetics with the occurrence of senescence in MSCs. The alteration of mtDNA methylation is accompanied by the upregulation of COX1 gene in both senescent MSCs and the DNMT-knocked down MSCs. This study thus implicates the mtDNA epigenotype as a critical biomarker in cellular senescence of MSCs.

## Supplementary Material

File 1: The ECM pathway in senescent MSCs. File 2: The AKT pathway in senescent HMSCs. File 3: mtDNA methylation of other CpG sites in senescent HMSCs. File 4: mtDNA methylation of other CpG sites in senescent SMSCs. File 5: mtDNA methylation of other CpG sites in old fibroblasts. File 6: PCR oligonucleotide primers.

## Abbreviations

MSCs: Mesenchymal stem cells
mtDNA: Mitochondrial DNA
DNMT: DNA methyltransferase
CpG: Regions of DNA where a cytosine nucleotide is followed by a guanine nucleotide separated by only one phosphate.
