# Supplementary material for: Mitochondrial DNA Hypomethylation Is a Biomarker Associated with Induced Senescence in Human Fetal Heart Mesenchymal Stem Cells
Source: Stem Cells Int. 2017 Apr 6;2017:1764549. doi: 10.1155/2017/1764549 (PMC5397648; doi:10.1155/2017/1764549)
Supplement: Supplementary file 1 — File 1: The ECM pathway in senescent MSCs. File 2: The AKT pathway in senescent HMSCs. File 3: mtDNA methylation of other CpG sites in senescent HMSCs. File 4: mtDNA methylation of other CpG sites in senescent SMSCs. File 5: mtDNA methylation of other CpG sites in old fibroblasts. File 6: PCR oligonucleotide primers. [file 1764549.f1.pdf]

Mitochondrial DNA hypomethylation is a biomarker associated with induced senescence in human fetal heart mesenchymal stem cells

Dehai Yu[1,2], Zhonghua Du[1,2], Lingling Pian[1,2], Tao Li[2], Xue Wen[1], Wei Li[1], Su-Jeong Kim[3], Jialin Xiao[3], Pinchas Cohen[3*], Jiuwei Cui[1*], Andrew R. Hoffman[2*], Ji-Fan Hu[1,2*]

[1] Stem Cell and Cancer Center, First Affiliated Hospital, Jilin University, Changchun, Jilin 130061, P.R. China

[2] Stanford University Medical School, Palo Alto Veterans Institute for Research, Palo Alto, CA 94304, USA

[3] Leonard Davis School of Gerontology, University of Southern California, Los Angeles, CA 90089, USA

SUPPLEMENTARY FIGURES

FIGURE LEGEND

Figure S1. The ECM pathway in senescent MSCs cultured from human fetal heart tissues (HMSCs)**.** Total RNAs were isolated from HMSCs for RNA-Seq using a HiSeq4000 (Illumina). KEGG pathways were selected as significantly regulated if the corrected p-values were < 0.05. Genes highlighted with boxes represent up-regulated (red) and down-regulated (green) during replicative senescence based on normalized FPKM values for each gene.

Figure S2. The AKT pathway in senescent HMSCs. After RNA-Seq using a HiSeq4000 (Illumina), KEGG pathways were selected as significantly regulated if the corrected p-values were < 0.05. Genes highlighted with boxes represent up-regulated (red) and down-regulated (green) during replicative senescence based on normalized FPKM values for each gene.

Figure S3. Mitochondrial DNA methylation of other CpG sites in senescent MSCs cultured from human fetal heart tissues (HMSCs, 8 CpG islands).

A. Schematic diagram of the mitochondrial genes and the location of CpG islands.

B. Comparison of mtDNA methylation between the control and senescent HMSCs. Cellular senescence was induced by exposing HMSCs to 50 µM H_2O_2 and 5% FBS in the medium, and mtDNA methylation was measured by combined

bisulfite restriction analysis (COBRA). PCR products from mtDNA of control and senescent HMSCs were digested by TaqI or HpyCH4IV (HPY) to distinguish the unmethylated and methylated DNAs, and were separated on 3% agarose gels. Only the data of CpG islands 3, 5-8 are presented here.

Figure S4. Mitochondrial DNA methylation other CpG sites in senescent MSCs cultured from human fetal skin tissues (SMSCs, 8 CpG islands).

A. Schematic diagram of the mitochondrial genes and the location of CpG islands.

B. Comparison of mtDNA methylation between the control and senescent SMSCs. Cellular senescence was induced by exposing SMSCs to 50 μM H_2O_2 and 5% FBS in the medium, and mtDNA methylation was measured by combined bisulfite restriction analysis (COBRA). PCR products from mtDNA of control and senescent HMSCs were digested by TaqI or HpyCH4IV (HPY) to distinguish the unmethylated and methylated DNAs, and were separated on 3% agarose gels. Only the data of CpG islands 3, 5-8 are presented here.

Figure S5. Differential mtDNA methylation other CpG sites between the human neonatal and adult fibroblasts (8 CpG islands).

A. Schematic diagram of the mitochondrial genes and the location of CpG islands.

B. Differential mtDNA methylation between neonatal and adult fibroblasts. mtDNA methylation was measured by combined bisulfite restriction analysis (COBRA). PCR products from mtDNA of neonatal and adult fibroblasts were digested by TaqI or HpyCH4IV (HPY) to distinguish the unmethylated and

methylated DNAs, and were separated on 3% agarose gels. Only the data of CpG islands 3, 5-8 are presented here.

SUPPLEMENTARY TABLES

Table S1. PCR oligonucleotide primers for CpG islands 1-11.

CpG island	Oligo Name	Oligo sequence	Product Size
1	JH2970	ACGGAATAAGTTATTTTAGGGATAAT	**224bp**
	JH3046	ACGAACCTTTAATAACGACTACACCA	
2	JH3048	CGAATTAAACCAAAAAATTAATTAATAC	**185bp**
	JH2974	AGGAGTTTAAATTTTTTTATTTTTAGGAT	
3	JH3049	AACCTCTTTTTACCAACTCCGAAATA	**147bp**
	JH2976	AATAGTT AAGTATTTTA ATTAATTGGT	
4	JH3050	AAACCTACAAATAATAAAATATTTCATA	**180bp**
	JH2978	TTTATTTTTAGGTTATATTTTAGATTAA	
5	JH3052	GGTTTAATATTTTTTGTAGTTATAGGT	**169bp**
	JH2983	ATCAAACCACATCTACAAAATACCAA	
6	JH3055	ACTATTTATTATAAATCTCATAAATTAA	**165bp**
	JH2988	AGGAATTATATTTTTTTTGTTTATTAG	
7	JH3057	GTTGTATTTTAATTATAAGAATATTAATG	**169bp**
	JH2993	CCTATAATAATTTAAAAAATCAAACAA	
8	JH3058	TATTAGATTTTTTAGGCGATTTAGATA	**138bp**
	JH2997	AAACGCCTCCTAATTTATTAA	
9	JH2972	TAATCGTAATGGTATTTTTAATGT TTAT	**141bp**
	JH3098	AAAACTCTTTAATAAAAAATTTTATAAC	
10	JH3056	TAGGTTAATTTCGTTTTTTTATTTTTAT	**124bp**
	JH3099	AAAATATTATTAATAATAAAAAATCCTAC	
11	JH2994	ATAGGATTATTTTTAGTTATGTAT	**214bp**
	JH2995	TAATACCGATATTTCAAATTTCTAAA	

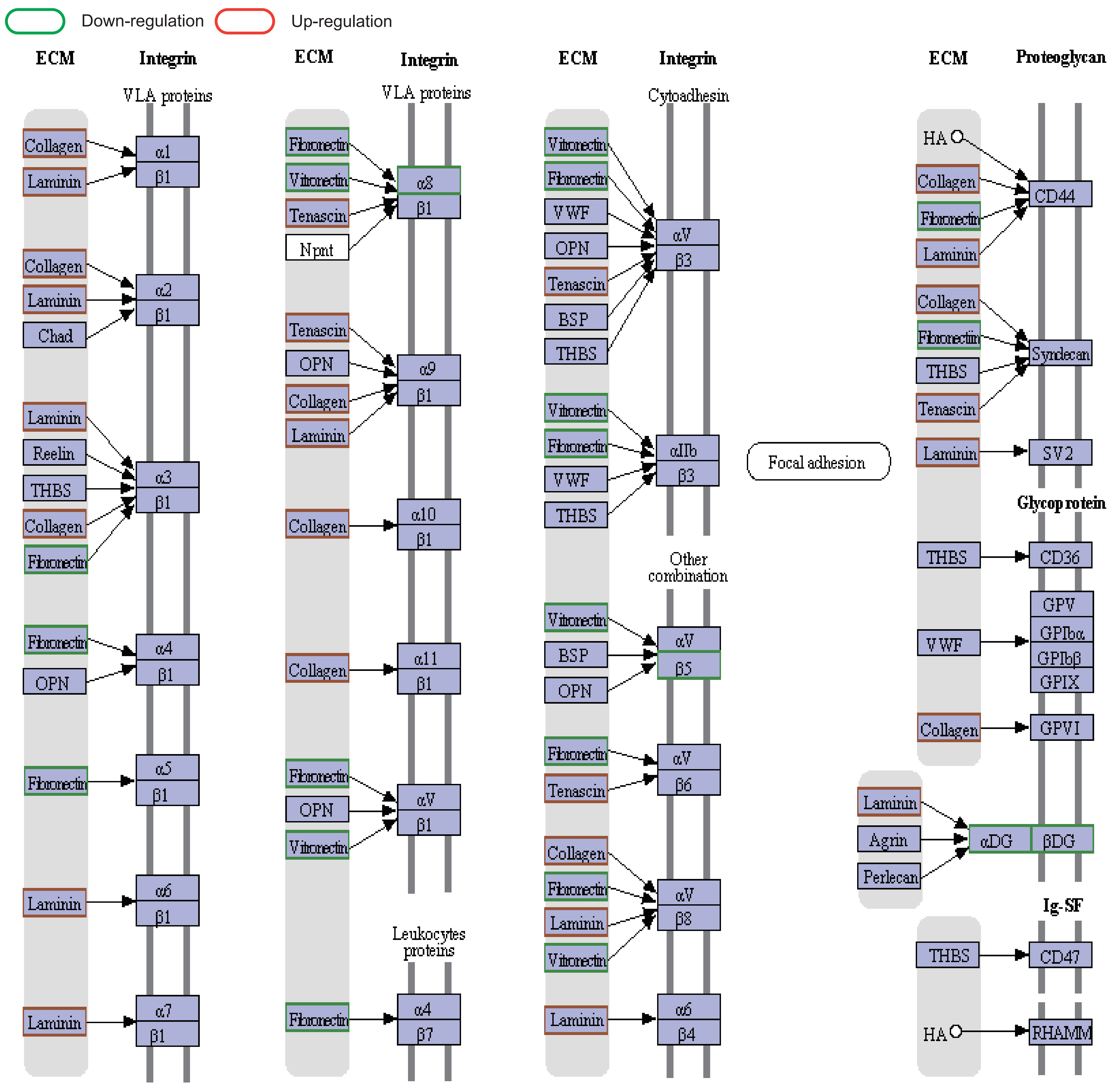

Gene ID	logFC(AgMSC/HdMSC)	q-value<0.05
FN1	-15.8819477	1.61E-14
ITGB5	-8.79907284	0.000287829
COL5A1	-7.97665592	0.003505373
VTN	-6.06076155	2.08E-05
ITGA8	-5.80522286	3.52E-06
TNXB	-5.54790327	3.75E-06
COL3A1	-4.86197037	3.65E-05
LAMA1	-3.45255312	0.004010821
LAMA5	-3.12243094	0.010891277
DAG1	-3.09489957	0.0220609
COL4A4	-3.05444632	0.016974287
LAMA4	2.75972297	0.030525022
COL4A5	3.617758	0.002506254
COL5A3	3.66121947	0.002134789
LAMA4	3.66914049	0.002016458
COL6A3	5.98034444	1.77E-06
TNC	7.4373014	1.48E-08

Figure S1. Activation of the ECM pathway in senescent HMSCs

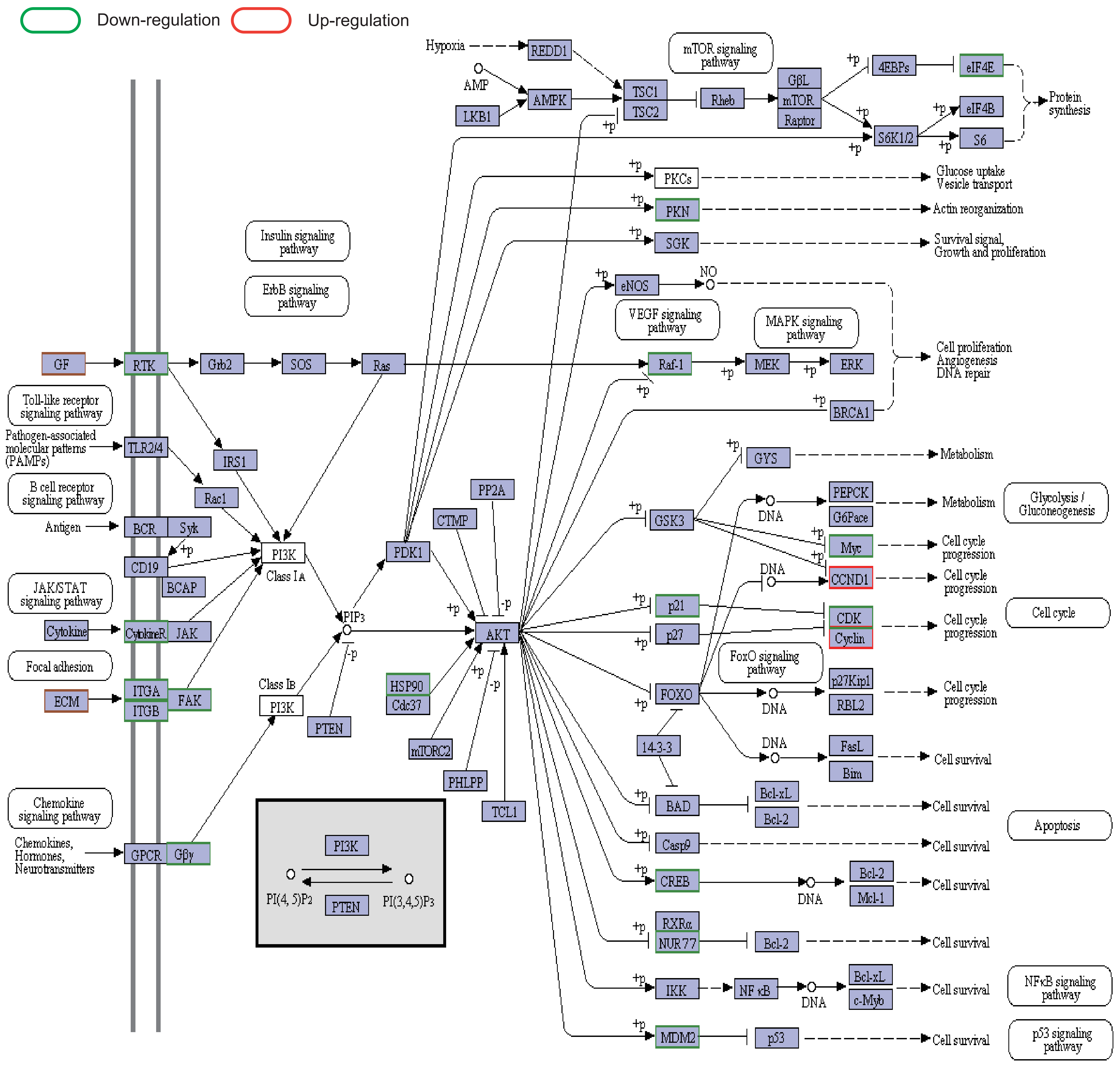

Gene ID	logFC(AgMSC/HdMSC)	q-value(<0.05)
ANGPT1	4.719789	6.98E-05
ATF2	-8.84478	0.00023981
CCND1	3.394681	0.010090873
CDK4	-3.93327	0.003928549
CDKN1A	-9.94113	6.15E-06
COL3A1	-4.86197	3.65E-05
COL4A4	-3.05445	0.016974287
COL4A5	3.617758	0.002506254
COL5A1	-7.97666	0.003505373
COL5A3	3.661219	0.002134789
COL6A3	5.980344	1.77E-06
EIF4E	-8.57335	0.000568414
FGF7	2.841623	0.026383721
FGFR2	-10.3266	1.74E-06
FN1	-15.8819	1.61E-14
GHR	-3.99299	0.002220827
GNB2	-9.58498	2.01E-05
HSP90AB1	-9.4875	2.85E-05
ITGA8	-5.80522	3.52E-06
ITGB5	-8.79907	0.000287829
LAMA1	-3.45255	0.004010821
LAMA4	3.66914	0.002016458
LAMA5	-3.12243	0.010891277
MDM2	-9.36738	4.18E-05
MYC	-11.7124	1.98E-08
NGF	-5.04044	0.004745608
NR4A1	-8.23874	0.001497132
PKN1	-11.1184	1.19E-07
PTK2	-2.96042	0.029835517
RAF1	-3.16464	0.025945288
TNC	7.48518	8.77E-09
TNXB	-5.5479	3.75E-06
VEGFA	-11.3891	5.43E-08
VTN	-6.06076	2.08E-05

Figure S2. Activation of the AKT pathway in senescent HMSCs

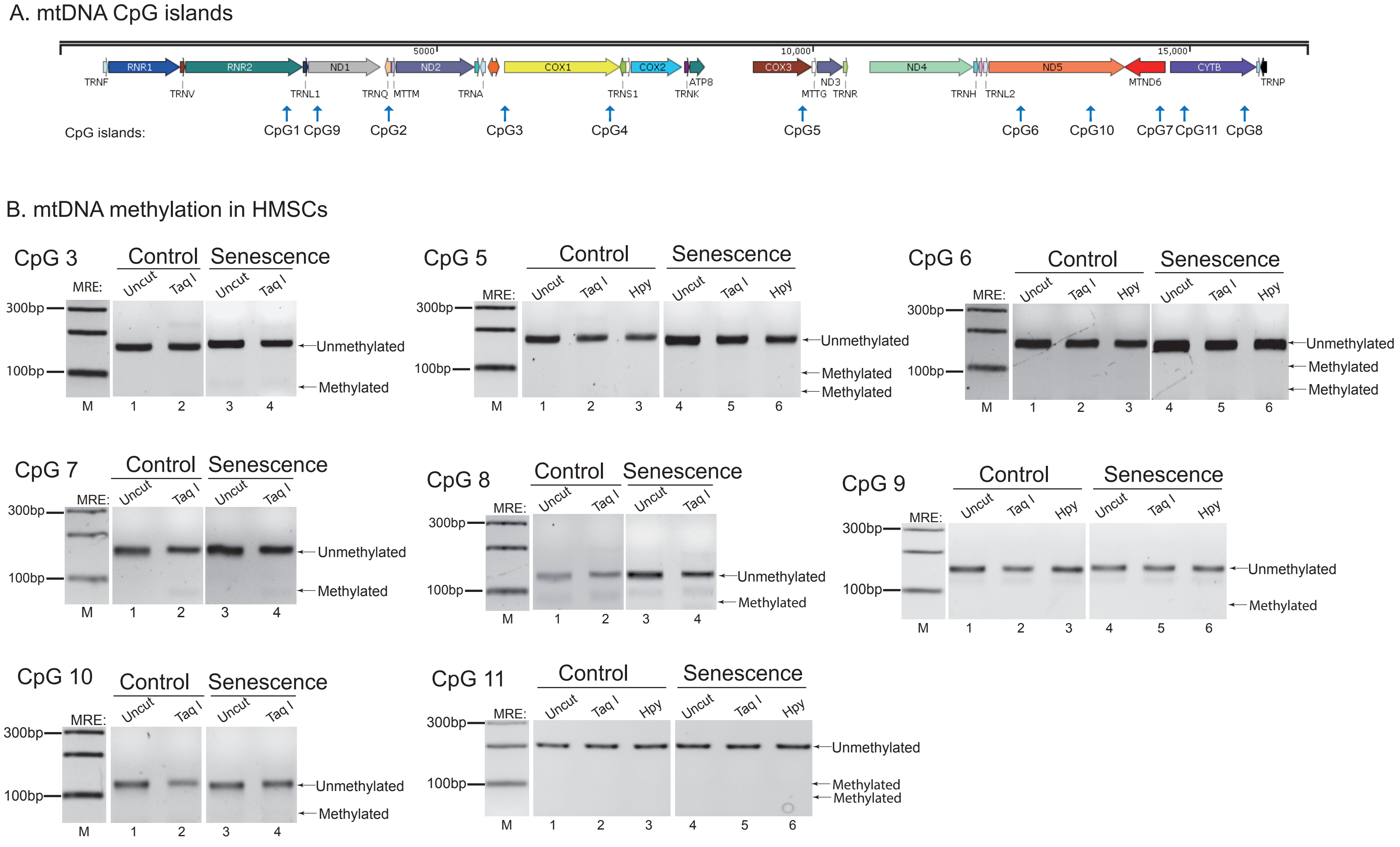

Figure S3. mtDNA methylation in senescent HMSCs

A. mtDNA CpG islands

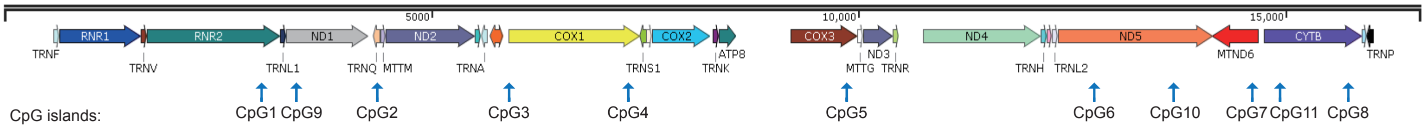

B. mtDNA methylation in HMSCs

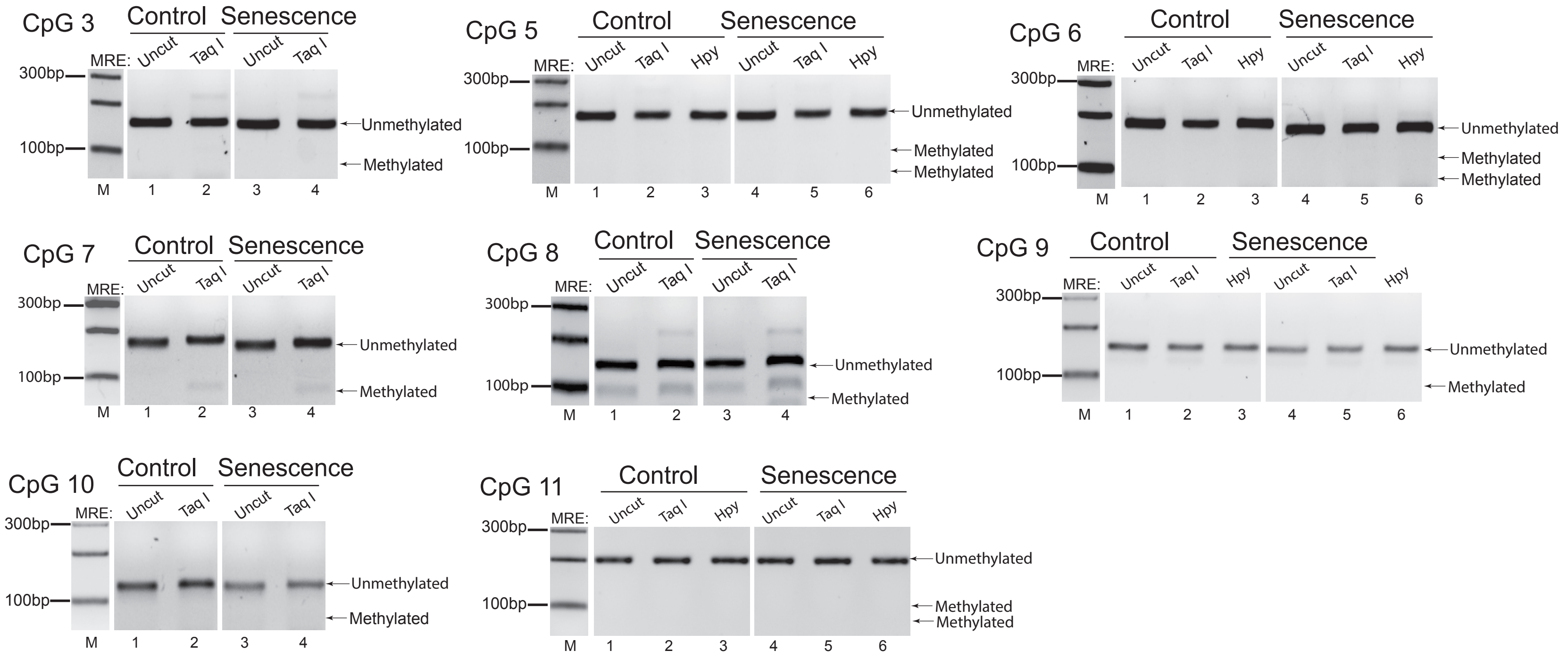

Figure S4. mtDNA methylation in senescent SMSCs

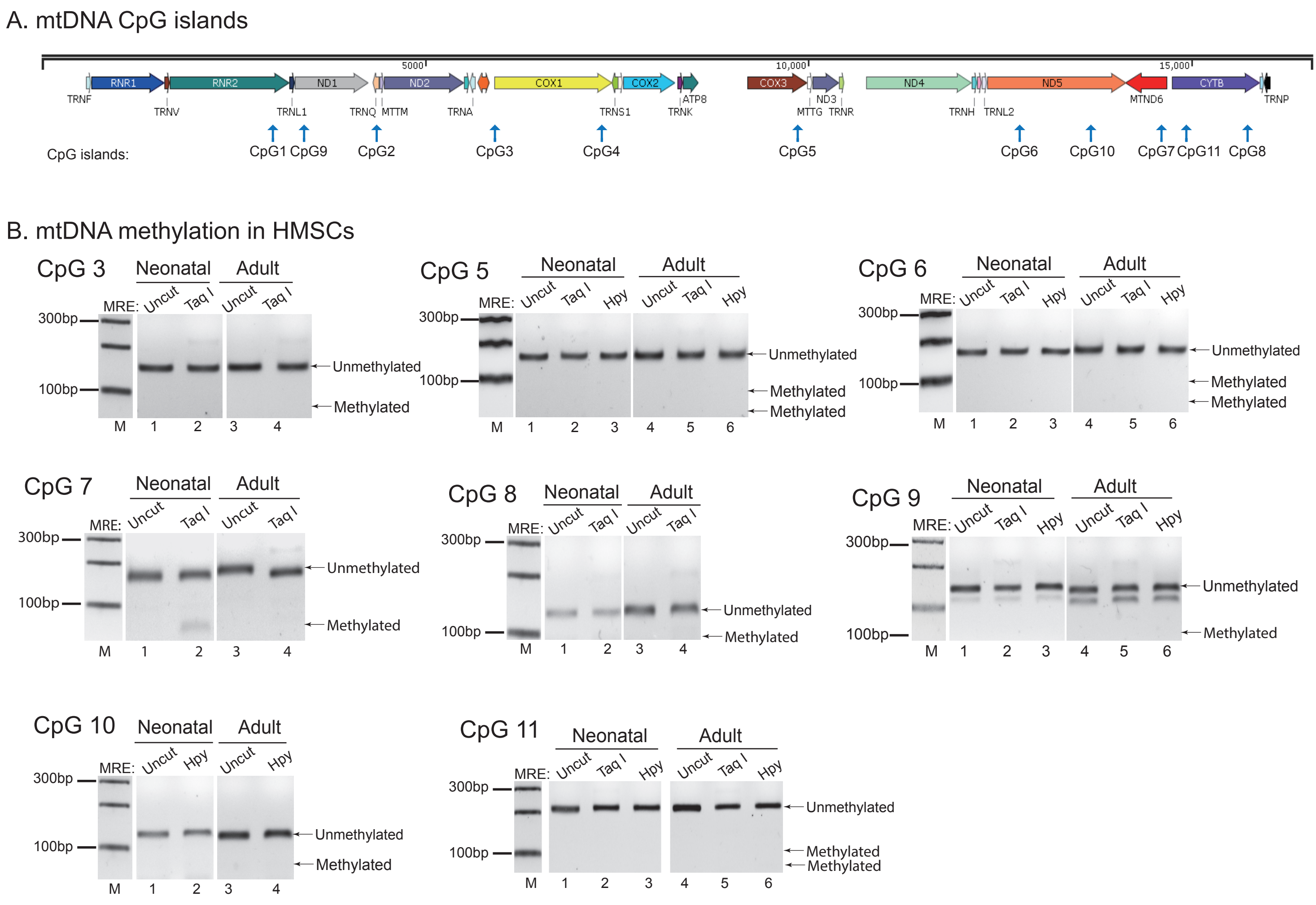

Figure S5. mtDNA methylation in neonatal and adult fibroblasts
